# Transforming Acute Myeloid Leukemia Treatment Through Next-Generation Sequencing: A Single-Center Experience

**DOI:** 10.7759/cureus.45917

**Published:** 2023-09-25

**Authors:** Vishal Devarkonda, Hugo Akabane

**Affiliations:** 1 Internal Medicine, Louisiana State University Health Sciences Center, Shreveport, USA; 2 Hematology and Medical Oncology, Louisiana State University Health Sciences Center, Shreveport, USA

**Keywords:** acute leukemia, chemoradiotherapy (chemo-rt), acute myeloid leukemia (aml), leukemia, acute myeloblastic leukemia

## Abstract

In the last decade, advancements in understanding the genetic and molecular mechanisms of acute myeloid leukemia (AML) have significantly improved treatment options. Techniques such as immunophenotyping, cytogenetics, and next-generation sequence (NGS) testing are now standard practices for patient assessments, allowing for personalized therapies based on individual patient needs. Our study aimed to evaluate the impact of cytogenetics and NGS on initial treatment decisions for AML at our institution. We analyzed the frequency of alternative therapy choices that could have been made with complete molecular and cytogenetic information and compared overall survival rates between patient groups. We also analyzed the turnaround time for result generation. Our retrospective study evaluated 39 AML patients treated at our university hospital from June 2020 to June 2022, excluding classic acute promyelocytic leukemia cases. Patients with incomplete data or concurrent hematological malignancies were excluded. We collected data on admission blood counts, European LeukemiaNet (ELN) risk stratification, Charlson score, treatment type, and timing of cytogenetics and NGS results. Patients were categorized into 'standard' or 'other therapy' groups based on their molecular and cytogenetic profiles in accordance with NCCN guidelines. Our main goal was to determine how often NGS and cytogenetics results could have influenced induction therapy choices. Secondary objectives included comparing overall survival rates and analyzing report turnaround times for NGS and cytogenetics. Our study found that out of the 39 AML patients, 17 were in the "standard" group, and 22 were in the "other therapy" group. The standard group had an average age of 62.59 years, an average time to chemotherapy initiation of 8.29 days, and an overall survival (OS) rate of 428.12 days. The other therapy group had an average age of 61.86 years, an average time to chemotherapy initiation of six days, and an OS rate of 258.64 days. There was a significant difference in survival rates between the two groups (p=0.009). According to the ELN stratification, the standard group had 11 patients at intermediate risk and six at adverse risk. In contrast, the other therapy group had seven at intermediate risk, four at good risk, and 11 at adverse risk. NGS revealed mutations in 58.97% of patients. Our study suggests that almost half of the patients could have been treated differently if complete molecular and cytogenetic information had been available before therapy initiation, highlighting the potential for more personalized treatments. Additionally, our results showed significant differences in overall survival rates between standard treatment and alternative therapy groups. Our findings emphasize the importance of timely NGS and cytogenetics result generation, guiding institutions to allocate resources for effective patient care.

## Introduction

Significant advancements have been made in treating acute myeloid leukemia (AML) in the last 10 years. Our increased understanding of the disease's genetic and molecular mechanisms has been crucial in this progress. These breakthroughs have led to the creation of innovative therapies and the integration of new biomarkers into routine patient assessments [1]. As a result, hematologists must adapt to a changing landscape where modern molecular techniques can provide essential supplementary information. The European Leukemia Net (ELN) recommends immunophenotyping, cytogenetics, and next-generation sequencing (NGS) testing for all newly diagnosed AML patients to ensure a comprehensive evaluation. These additional data enable more accurate risk stratification and sub-classification of AML [1]. In managing AML, it's becoming increasingly important to identify patient subgroups that may benefit from customized treatments. Studies have shown that patients with FLT3 Internal Tandem Duplication (ITD) and Tyrosine Kinase Domain (TKD) mutations, favorable-risk leukemias, and therapy-related AML (t-AML), or AML with myelodysplasia-related changes (AML-MRC) can benefit from personalized therapy [2-4]. This highlights the need to uncover previously unknown mutations that could lead to new molecular pathways and targetable mutations. Participating in clinical trials or receiving unconventional therapy may be beneficial for many patients [2]. The usual treatment for AML is intensive chemotherapy, but it has a high mortality risk and morbidity risk. This type of treatment needs to be started quickly, which makes it difficult to incorporate genomic data into the initial therapy decisions. However, the Beat AML trial (ClinicalTrials.gov NCT03013998) has proven that a faster precision medicine therapy approach is possible without worsening early outcomes [5]. Our study aimed to investigate how cytogenetics and NGS affect the initial treatment of newly diagnosed AML patients at our medium-sized university hospital. We wanted to determine how often treating physicians would choose alternative therapy if all the molecular and cytogenetics studies were available. We also looked at the differences in overall survival between patients who received these tests upfront and those who did not. Additionally, we documented the time needed to generate all the results. To categorize patients whose treatment plans might have been changed if cytogenetics and NGS outcomes were available upfront, we used the National Comprehensive Cancer Network (NCCN) decision-making algorithm and the treating physician's discretion. Our goal was to explore the effect of these genetic testing methods on AML treatment decisions and patient outcomes. By providing valuable insights, our findings will help healthcare providers make informed clinical decisions and improve patient outcomes in AML management. Our research is especially relevant for smaller healthcare establishments that may need help in procuring NGS results quickly due to equipment, geographical location, or personnel constraints. Ultimately, our study will help determine the feasibility of using genomic data to inform initial therapy decisions and assess the impact of timely testing on patient outcomes [5-6].

## Materials and methods

We conducted a retrospective evaluation of 39 patients diagnosed with AML who had undergone treatment at our university hospital between June 2020 and June 2022. Our inclusion criteria comprised patients diagnosed with AML, who underwent treatment at our university hospital during the specified period. We excluded patients with incomplete or inadequate data for examination and patients with concurrent or mixed diagnoses of acute leukemia, classic acute promyelocytic leukemia, and other hematological malignancies.

We collected a variety of data points for each patient, including admission blood counts, ELN risk stratification, Charleston score, treatment type, time to cytogenetics results, time to NGS results, therapy response, MRD status, therapy initiation type, and allogeneic stem cell transplant consolidation. After gathering data, we utilized the most recent National Comprehensive Cancer Network (NCCN) guidelines for AML to identify patients with different treatment options based on their molecular and cytogenetic profiles. We presented each patient's case to the attending physician, asking whether they would have suggested another or similarly effective treatment with less probable toxicity based on the molecular and cytogenetic results. Patients whose treatment would have differed according to these guidelines were classified as "other therapy" while those whose treatment would not have varied were classified as "standard." We examined all the data points, with a specific emphasis on molecular and cytogenetic changes, such as FLT3 ITD/TKD, cytogenetic changes linked with myelodysplasia, tp53 mutation, complex karyotype, genetic mutations associated with MDS, RUNX1-RUNX1T1, CBFB-MYH11, Variant-type PML/RARA, BCR/ABL, IDH1/2, NPM1, CEBPA, and MLL genes. Before data collection, the local ethics committee approved the study, and all patients were assessed in the group to which they were assigned. We estimated right-censored data using the Kaplan-Meier method and compared it using the log-rank test. We collected relevant variable means and reported them in the results section.

The study aimed to assess the impact of NGS and cytogenetics on the induction therapy of newly diagnosed AML patients. The study’s primary objective was to determine how frequently NGS and cytogenetics results could have influenced the attending physician's choice of induction therapy. As a secondary objective, we compared overall survival between the two groups. We defined overall survival as the time from diagnosis to death. We also analyzed data on the time to report results from NGS and cytogenetics. NGS assessed 42 genes and selected intronic regions, including ANKRD26, ASXL1, BCOR, CALR, CBL, CEBPA, CSF3R, DDX41, DNMT3A, ELANE, ETNK1, ETV6, EZH2, FLT3, GATA1, GATA2, IDH1, IDH2, JAK2, KDM6A, KIT, KRAS, MPL, NPM1, NRAS, PHF6, PTPN11, RAD21, RUNX1, SETBP1, SH2B3, SF3B1, SRP72, SMC3, SRSF2, STAG2, TERT, TET2, TP53, U2AF1, WT1, and ZRSR2. Additional BCR/ABL amplification by polymerase chain reaction (PCR) was requested for all patients.

## Results

**Figure 1:**
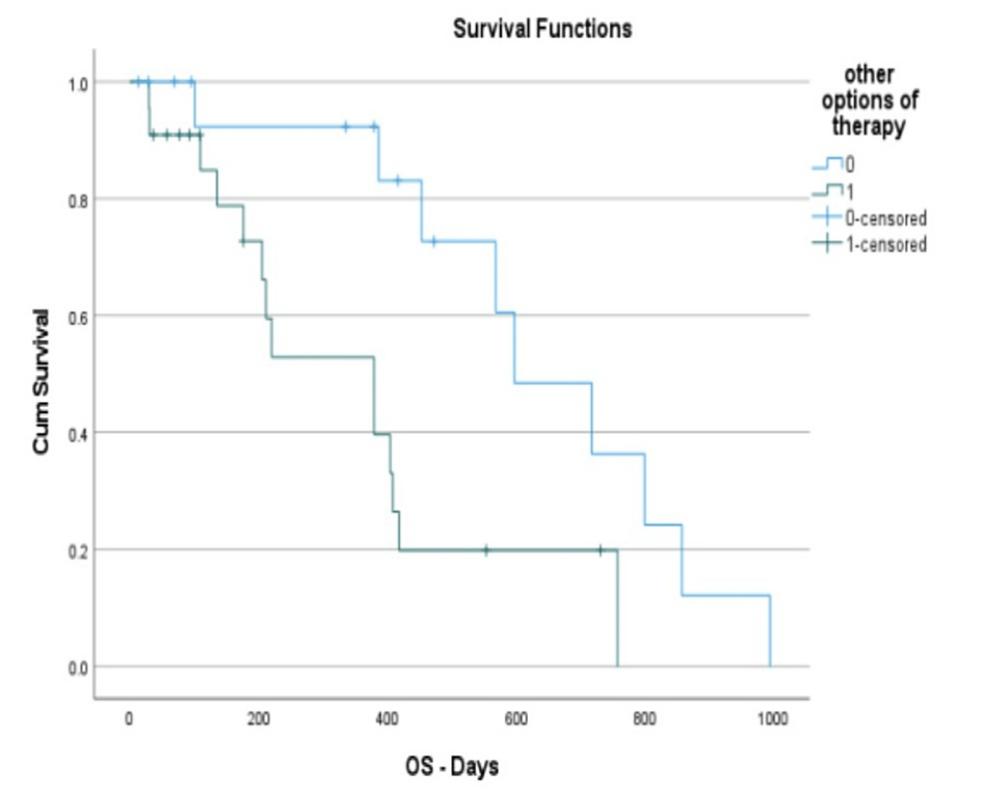
Comparing cumulative survival: standard vs. other therapy with days of overall survival on the x-axis and cumulative survival rate on the y-axis, highlighting the standard group’s survival advantage Legend: 0 (Standard Group) and 1 (Other Group)

**Figure 2:**
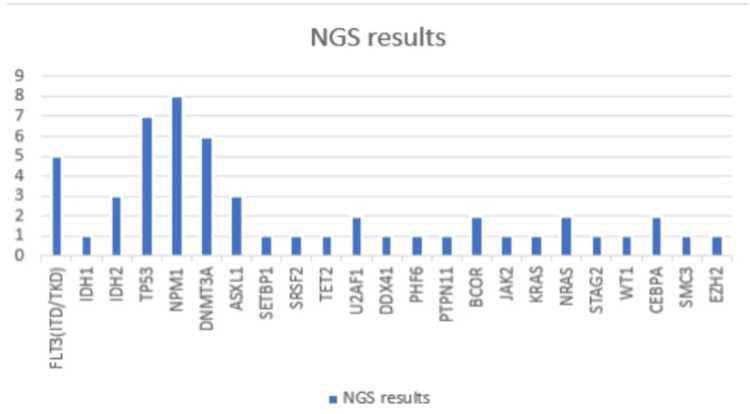
Genetic distribution across patients with x-axis: cumulative NGS results for all patients and y-axis: number of patients with certain genes FLT3 - FMS-like tyrosine kinase 3 (FLT3), IDH1 - Isocitrate dehydrogenase 1 (IDH1), IDH2 - Isocitrate dehydrogenase 2 (IDH2), TP53 - Tumor protein p53 (TP53), NPM1 - Nucleophosmin 1 (NPM1), DNMT3A - DNA methyltransferase 3A (DNMT3A), ASXL1 - Additional sex combs like 1 (ASXL1), DDX41 - DEAD-box RNA helicase 41 (DDX41), CEBPA - CCAAT/enhancer-binding protein alpha (CEBPA), PHF6 - PHD finger protein 6 (PHF6), PTPN11 - Protein tyrosine phosphatase non-receptor type 11 (PTPN11), SETBP1 - SET binding protein 1 (SETBP1), SRSF2 - Serine/arginine-rich splicing factor 2 (SRSF2), U2AF1 - U2 small nuclear RNA auxiliary factor 1 (U2AF1), SMC3 - Structural maintenance of chromosomes protein 3 (SMC3), BCOR - BCL6 corepressor (BCOR), JAK2 - Janus kinase 2 (JAK2), NRAS - Neuroblastoma RAS viral oncogene homolog (NRAS), STAG2 - Stromal antigen 2 (STAG2), WT1 - Wilms tumor 1 (WT1), EZH2 - Enhancer of zeste homolog 2 (EZH2), KRAS - Kirsten rat sarcoma viral oncogene homolog (KRAS), TET2 - Ten Eleven Translocation-2 (TET2)

The study analyzed data from 39 participants, with 17 assigned to the "standard" group and 22 allocated to the "Other therapy" group. In the "standard" group, The mean age of diagnosis was 62.59 ± 13.77 years. Hemoglobin levels averaged 8.51 ± 1.74 g/dL while the blast count in peripheral blood exhibited a mean of 23.82 ± 33.23 ×10^9/L. The white blood cell count was 8.06 ± 17.87 ×10^9/L, and platelet counts averaged 69 ± 50.38 ×10^9/L. The Charlson Comorbidity Index had a mean value of 5.51 ± 2.47. The time to initiate therapy was on average 7.00 ± 5.45 days, with a waiting time of 12.71 ± 4.26 days for NGS results and 7.31 ± 3.29 days for cytogenetics results. In the Standard group, four patients underwent high-intensity chemotherapy, and 13 underwent low-intensity chemotherapy. The overall survival (OS) had a mean of 428.12 ± 302.98 days. In comparison to the "Other group"(Refer to Table 1), the average age of diagnosis was 61.86 ± 14.65 years. Hemoglobin levels were found to be higher, with a mean of 9.15 ± 2.68 g/dL. In terms of blast count in peripheral blood, this group exhibited an average of 32.29 ± 31.79 ×10^9/L. The white blood cell count was notably elevated, with a mean of 35.95 ± 63.86 ×10^9/L, and platelet counts averaged 96.23 ± 92.57 ×10^9/L. The Charlson Comorbidity Index had a mean value of 5.14 ± 2.189 in this subgroup. The time to initiate therapy was slightly lower compared to the overall cohort, with an average of 6.00 ± 5.39 days. The waiting time for NGS results was 13.41 ± 4.18 days, and the waiting time for cytogenetics results was 6.86 ± 3.04 days. Regarding the OS, the mean was 258.64 ± 217.41 days. Thirteen patients underwent 22 high-intensity chemotherapies, and nine underwent low-intensity chemotherapy. A statistically significant difference in survival was observed between the two groups (p=0.009) (Figure 1). In the standard group, 11 patients were at ELN intermediate risk, zero at good risk, and six at adverse risk. In the "other therapy" group, seven patients were at ELN intermediate risk, four were at good risk, and 11 were at adverse risk. As per the results of the NGS, 58.97% of patients exhibited findings: Five displayed FLT3 mutations, one IDH1, three IDH2, seven P53 mutations, eight NPM1, six DNMT3A, three ASXL1, one DDX41, two CEBPA, one PHF6, one 23PTPN11, one SETBP1, one SRSF2, two U2AF1, one SMC3, two BCOR, one JAK2, two NRAS, one STAG2, one WT1, and one EZH2. The outcomes of all patients' NGS are exhibited in Figure 2. The primary rationale for assigning patients to the "other therapy" group included: the presence of genes associated with myelodysplastic syndrome (14), tp53 (5), RUNX1-RUNX1T1 (3), CBFB-MYH11 (1), cytogenetics indicating myelodysplastic changes (10), variant-type PML/RARA (1), MLL (1), and FLT3 (3). Notably, individual patients could have multiple reasons for their assignment to the "other therapy" group. 

**Table 1:** Demographic and clinical characteristics of the standard group (n=17) in comparison with the other group (n=22)

Characteristics	Standard Group Mean ± SD	Other Group Mean ± SD
Age of diagnosis	62.59 ± 13.77 years	61.86 ± 14.65 years
Hemoglobin	8.51 ± 1.74 g/dL	9.15 ± 2.68 g/dL
Blast count on peripheral blood	23.82 ± 33.23 ×10^9/L	32.29 ± 31.79 ×10^9/L
White blood count	8.06 ± 17.87 ×10^9/L	35.95 ± 63.86 ×10^9/L
Platelets	69 ± 50.38 ×10^9/L	96.23 ± 92.57 ×10^9/L
Charlson Comorbidity Index	5.51 ± 2.47	5.14 ± 2.189
Time to start therapy	7.00 ± 5.45 days	6.00 ± 5.39 days
Waiting time for NGS results	12.71 ± 4.26 days	13.41 ± 4.18 days
Waiting time for cytogenetics	7.31 ± 3.29 days	6.86 ± 3.04 days
OS (Overall Survival)	428.12 ± 302.98	258.64 ± 217.41

## Discussion

Our study, which analyzed data from 39 AML patients, aimed to investigate the impact of delaying therapy initiation to await NGS and cytogenetics results on treatment decisions and patient outcomes. In the "standard" group, characterized by immediate therapy initiation, we observed an average overall survival (OS) of 428.12 days while the "other therapy" group, where treatment initiation was delayed for NGS and cytogenetics results, had an OS of 258.64 days. This difference was statistically significant (p=0.009) (Figure 1).

Our findings raise important questions about the optimal timing for treatment initiation in AML patients. Traditionally, early treatment was considered essential to prevent disease progression and improve survival. However, recent studies, including our own, suggest that delaying treatment to obtain molecular and cytogenetics information may not necessarily lead to worse outcomes, challenging the conventional wisdom [7].

To contextualize our results, we can draw upon existing research. Bertoli et al. conducted a single-center study in 2013 involving 205 AML patients and found no significant association between the time from diagnosis to treatment (TDT) and patient outcomes [8]. This study suggests that there might be room for flexibility in treatment initiation, aligning with our observations.

Röllig et al. further explored the relationship between TDT and outcomes in a cohort of 2,200 newly diagnosed AML patients. Their findings indicated no significant impact of TDT on complete remission, early death, or overall survival, even after adjusting for known prognostic factors [9]. These results support the idea that waiting for genetic and laboratory test results may be a reasonable approach for clinically stable AML patients.

The AML master trial led by Burd et al. demonstrated the feasibility of providing genetic and cytogenetic data within seven days of sample receipt, allowing for treatment decisions based on genomic information [5]. This approach did not lead to increased early mortality or negatively impact overall survival. The Beat AML trial also endorsed the safety and feasibility of using dominant clones for treatment assignment, showcasing the potential of precision medicine in AML.

In our study, we found that 43.59% of patients might have received different treatment decisions if their treating physicians had access to all molecular and cytogenetics results before initiating therapy. While having these results before treatment initiation doesn't guarantee better outcomes, it suggests the potential for more tailored and efficient treatment planning.

However, it's important to acknowledge the potential disadvantages of delaying treatment for NGS and cytogenetics results. Treatment delays can lead to immediate adverse consequences such as leukostasis, infections, anemia, and worsening performance status. Patients may also experience heightened anxiety and post-traumatic stress due to treatment postponement. Delaying AML treatment for genetic insights offers several key advantages. It enables personalized therapies tailored to individual patients, reduces the risk of ineffective treatments and side effects, and optimizes care according to disease severity. Additionally, it improves transplant success rates, lowers the risk of disease relapse, and has the potential to enhance overall survival rates. This approach also promotes resource efficiency within healthcare systems and provides access to cutting-edge clinical trials. In summary, delaying AML treatment for genetic insights leads to more effective, tailored care with improved outcomes. Our study has limitations, including a small sample size of 39 AML patients, potential biases due to its retrospective design, and limited generalizability due to its single-center nature.

## Conclusions

In summary, our study highlights the potential benefits of awaiting next-generation sequencing (NGS) results before initiating treatment for acute myeloid leukemia (AML). By analyzing data from 39 AML patients, we found that having complete molecular and cytogenetic information prior to therapy could have led to different treatment choices for almost half of these patients. Moreover, there were significant differences in overall survival rates between the standard treatment and other therapy groups. This underscores the importance of timely NGS and cytogenetics result generation, prompting institutions to prioritize resource allocation for improved patient care.

## Appendices

**Table 2:** Gene abbreviations in the article

Gene Abbreviation	Gene Name
FLT3	FMS-like tyrosine kinase 3
IDH1	Isocitrate dehydrogenase 1
IDH2	Isocitrate dehydrogenase 2
TP53	Tumor protein p53 (TP53)
NPM1	Nucleophosmin 1 (NPM1)
DNMT3A	DNA methyltransferase 3A
ASXL1	Additional sex combs like 1 (ASXL1)
DDX41	DEAD-box RNA helipads 41
CEBPA	CCAAT/enhancer-binding protein alpha (CEBPA)
PHF6	PHD finger protein 6 (PHF6)
PTPN11	Protein tyrosine phosphatase non-receptor type 11 (PTPN11)
SETBP1	SET binding protein 1 (SETBP1)
SRSF2	Serine and arginine-rich splicing factor 2 (SRSF2)
U2AF1	U2 small nuclear RNA auxiliary factor 1 (U2AF1)
SMC3	Structural maintenance of chromosomes protein 3 (SMC3)
BCOR	BCL6 corepressor (BCOR)
JAK2	Janus kinase 2 (JAK2)
NRAS	Neuroblastoma RAS viral oncogene homolog (NRAS)
STAG2	Stromal antigen 2 (STAG2)
WT1	Wilms tumor 1 (WT1)
EZH2	Enhancer of zeste homolog 2 (EZH2)
KRAS	Kirsten rat sarcoma viral oncogene homolog (KRAS)
TET2	Ten eleven translocation-2
